# The effect of three environmental conditions on the fitness of cytochrome P450 monooxygenase-mediated permethrin resistance in *Culex pipiens quinquefasciatus*

**DOI:** 10.1186/1471-2148-9-42

**Published:** 2009-02-19

**Authors:** Melissa C Hardstone, Brian P Lazzaro, Jeffrey G Scott

**Affiliations:** 1 Department of Entomology, Comstock Hall, Cornell University, Ithaca, NY, USA

## Abstract

**Background:**

The evolution of insecticide resistance and persistence of resistance phenotypes are influenced by the fitness of resistance alleles in the absence of insecticide pressure. Experimental determination of fitness is difficult, but fitness can be inferred by measuring changes in allele frequencies in appropriate environments. We conducted allele competition experiments by crossing two highly related strains of *Culex pipiens quinquefasciatus *mosquitoes. One strain (ISOP450) was permethrin resistant (due to P450-mediated detoxification) and one was a susceptible strain. Allele and genotype frequencies were examined for 12 generations under three environmental conditions: standard laboratory, temephos exposure (an insecticide to which the P450 detoxification mechanism in ISOP450 confers no resistance and which is commonly used in mosquito control programs) and cold temperature stress (mimics the colder temperatures within the habitat of this mosquito).

**Results:**

A fitness cost was inferred for the P450 mechanism in the standard laboratory environment. A greater cost was associated with the temephos exposed environment, suggesting the temephos placed an additional stress on the P450 resistant mosquitoes. No observed cost was associated with the P450 resistance locus in the cold temperature environment, but there was a significant heterozygote advantage. In all environments the fitness of the resistant homozygotes was the lowest.

**Conclusion:**

The cytochrome P450-mediated permethrin detoxification resistance in *Cx. p. quinquefasciatus *can have an associated fitness cost in the absence of permethrin, relative to a susceptible allele. The strength of the cost varies depending on the environmental conditions. P450-mediated resistance is expected to decrease over time if the permethrin application is relaxed and to decrease at an even faster rate if permethrin is replaced with temephos. Additionally, these results indicate that a P450 resistance allele can persist (especially in heterozygotes) in colder temperatures and could potentially be carried into the *Culex pipiens *hybrid zone.

## Background

Fitness of insecticide resistance mechanisms (*i.e*., alleles) can impact the evolution of resistance in the field, as well as the persistence of the resistance phenotype in untreated environments [1]. Models of resistance evolution within a pest population are based on the premise that resistance mutations occur only rarely, but are strongly favored in insecticide treated environments. Conversely, if in the absence of insecticide selection pressures resistant individuals suffer a fitness cost, the frequency of the resistance allele will decrease and the population will revert towards susceptibility [2,3]. In the absence of insecticide, resistant insects have been found to be less successful at overwintering [4-6], mating [7], predator avoidance and parasite resistance [8-10], and general development [11]. If no cost is associated with the resistance allele in the absence of insecticide, then it is possible for the resistance allele to become fixed in a population.

Determining the fitness of an insecticide resistance allele [2] is difficult, but can be undertaken using allele competition experiments between genetically related strains [12]. A number of methods and variables are used to assess fitness of a resistance allele. One common approach tracks allele frequencies of a population either in an untreated environment through time [13-15] or at one time point over a transect that includes areas that are insecticide treated and untreated [13,16]. The former of these two types of studies can be conducted in the laboratory using population cages [1]. In population cage experiments, when allele frequencies decrease, increase or remain constant through time, they are considered costly, beneficial or neutral, respectively. Failure to detect a fitness cost could be due to fitness associated with the focal allele truly being beneficial or neutral, evolution of a compensatory mutation that mitigates the cost of the resistance allele [17], or the correlated antagonistic pleiotropy maybe undetectable (either because the physiological cost might only be manifest in specific environments other than those tested [18], or the cost may affect characters that are not examined [19]). A second type of methodology compares biological components of fitness, such as mating competition, longevity, and reproductive output, between populations of interest [1,11]. For either method, fitness costs or benefits are most meaningful when genetically related strains are used, and may be observed more easily if alleles are in competition with each other [20]. Fitness costs may be manifested under most conditions, or only under certain environments, such as when populations are at high densities or resources are poor or scarce [21].

Examining the fitness of a resistant pest species in different environments, including an insecticide-free environment, is important for creating successful and sustainable resistance management, for maintaining insecticide effectiveness by elucidating the dynamics of the resistance allele and for providing information for optimal timing of insecticide rotations [22]. We conducted a laboratory population cage study using genetically related strains of the mosquito *Culex pipiens quinquefasciatus *(SLAB and ISOP450) subjected to environmental conditions that are relevant to the type of stresses this mosquito species could be exposed to in the field. Altering the environmental stresses in a laboratory setting allowed us to avoid confounding variables (such as food availability, larval development space, and differences in microclimates) that could also act on field populations of mosquitoes.

*Cx. p. quinquefasciatus *are important vectors of pathogens that infect both humans and other animals. This species is the primary vector of the filarial nematode *Wuchereria bancrofti *[23]. It is also capable of transmitting West Nile virus (WNV) [24,25], bird malaria pathogens, dog heartworm (*Dirofilaria immitis*), avian pox virus, and St. Louis encephalitis in the United States [26]. Insecticides are the major tool for mosquito control, therefore insecticide resistance is an important and ever increasing problem in control efforts [27].

Pyrethroid resistance in the *Culex pipiens *complex has been recorded around the world [28-38]. Resistance to pyrethroid compounds in *Culex *mosquitoes is conferred by two major mechanisms, enhanced detoxification by cytochrome P450 monooxygenases [29] as well as target site insensitivity (*i.e*., a L1014F mutation in the voltage sensitive sodium channel called *kdr*) [39]. Previously, we isolated a permethrin resistant (1,300-fold) strain of *Cx. p. quinquefasciatus *(named ISOP450) which was >99.9% related to the susceptible lab strain SLAB. Permethrin resistance in ISOP450 is monofactorial and due solely to cytochrome P450-mediated detoxification [40].

We were interested in determining if, and how quickly, the P450 resistance allele frequency would change when it was placed in competition with the susceptible allele under environmental stress. Our hypothesis was that under environmental stress the P450 detoxification resistance allele would be costly. Since this P450 detoxification mechanism was previously shown to be larval specific [40], measurements were conducted on larvae to see if associated costs were present. The environmental conditions imposed in the laboratory included insecticide-free standard rearing procedures, larval exposure to temephos, a commonly used organophosphate in mosquito control programs which is bioactivated (*i.e*., toxic metabolite is produced from the parent insecticide) by the P450 resistance mechanism [40], as well as larval exposure to a cold temperature (16°C) which represents the northern boundary for *Cx. p. quinquefasciatus *in its native geographic range. This experimental regime allowed us to examine the fitness associated with the cytochrome P450-mediated permethrin resistance mechanism in ISOP450 (the same mechanism present in the highly permethrin resistant JPAL strain [29]) in relation to known environmental stresses, as well as providing a greater understanding of resistance evolution when insecticide selection is relaxed.

## Results

**Figure 1:**
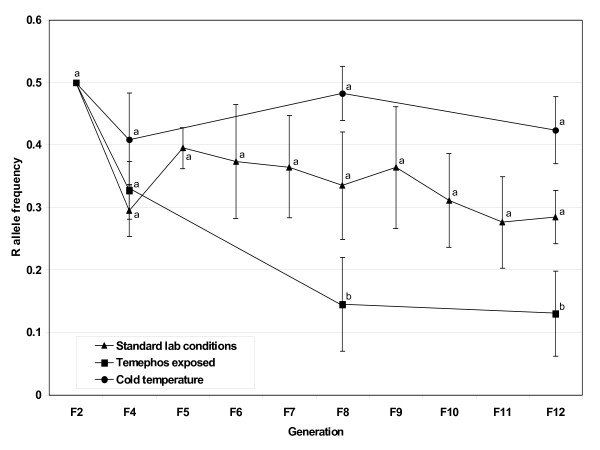
**P450 R allele frequency in three environmental conditions through 12 generations**. Frequencies of the P450-mediated detoxification R allele monitored through time in three environmental conditions: standard laboratory rearing, temephos exposure and cold temperature. Results are the averages of the four replicates for each environment. Bars are the S.E.M. Different letters indicate statistical differences (*p *< 0.05) between means.

Our results show that fitness costs are associated with the cytochrome P450-mediated permethrin detoxification resistance allele in *Cx. p. quinquefasciatus *(Figure 1) and that the strength of the cost varies depending on the environmental conditions. When fitness is assessed using resistant (R) allele frequencies, P450 detoxification is most costly in the temephos exposed environment, costly in the standard laboratory environment, and not costly in the cold temperature environment. Detailed results from each of the three environmental conditions are discussed below.

### Standard laboratory rearing conditions

**Figure 2:**
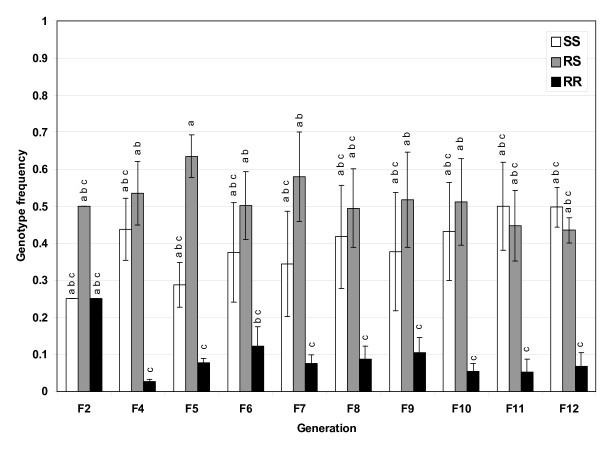
**P450 genotype frequencies in standard laboratory environment**. Frequencies of the P450-mediated detoxification genotypes monitored every generation for 12 generations in the standard laboratory environmental condition. Results are the average of four replicates. Bars are the S.E.M. Different letters indicate statistical differences (*p *< 0.05) between means.

In the absence of insecticide, the R allele frequency decreased from a starting frequency of 0.5 to 0.295 in F_12 _(Figure 1). While the frequency differences were not found to be statistically different due to the variation between the replicates (Tukey's HSD, *p *> 0.05), throughout the experiment the trend of a declining R allele frequency was steady with an exception at F_4 _where a larger dip occurred. Examination of the genotypes shows that the homozygous resistant genotype (RR) decreases, and for most generations stays below 0.1 (Figure 2). This trend was observed for both crosses (A and B) and for both replicates for each of these crosses, although there was some variation between generations (Table 1). This data indicates that the fitness cost of being RR is greater than the cost of having a single R allele in this environment. Interestingly, the RS genotype stays at a frequency of approximately 0.5, while the SS genotype tends to increase in frequency over the 12 generations (Table 1 and Figure 2). Frequency changes in the three genotypes ultimately leads to the trend of a decreasing frequency of the R allele and an increase in the S allele frequency.

**Table 1:** P450 genotype and allele frequencies in a standard laboratory environment

		**Genotype frequency** **Observed (Expected)**			**Allele Frequency**				
						
**Cross**	**Gen**.	**SS**	**RS**	**RR**	**S**	**R**	**n**^***a***^	**HWE**^***b***^	**Drift**^***b***^
									
A #1	F_2_	0.25	0.50	0.25	0.50	0.50			
	F_4_	0.69 (0.69)	0.28 (0.28)	0.03 (0.03)	0.83	0.17	120	0.9203	<0.0001*
	F_5_	0.31 (0.38)	0.62 (0.47)	0.07 (0.15)	0.62	0.38	960	<0.0001*	<0.0001*
	F_6_#	0.31 (0.34)	0.55 (0.49)	0.14 (0.17)	0.59	0.41	1040	<0.0001*	0.0105
	F_7_#	0.24 (0.33)	0.67 (0.49)	0.09 (0.18)	0.58	0.42	1560	<0.0001*	0.2039
	F_8_	0.19 (0.27)	0.66 (0.50)	0.15 (0.23)	0.52	0.48	900	<0.0001*	0.0004*
	F_9_#	0.17 (0.24)	0.63 (0.50)	0.20 (0.26)	0.49	0.51	1640	<0.0001*	0.0023
	F_10_#	0.21 (0.30)	0.68 (0.50)	0.11 (0.20)	0.55	0.45	1760	<0.0001*	1
	F_11_	0.24 (0.35)	0.70 (0.48)	0.06 (0.17)	0.59	0.41	1520	<0.0001*	0.0009*
	F_12_	0.47 (0.46)	0.41 (0.43)	0.12 (0.11)	0.68	0.32	400	0.19	0.0002*
									
A #2	F_2_	0.25	0.50	0.25	0.50	0.50			
	F_4_	0.35 (0.43)	0.61 (0.45)	0.04 (0.12)	0.66	0.34	80	0.0018	0.0024
	F_5_	0.44 (0.46)	0.48 (0.44)	0.08 (0.10)	0.68	0.32	420	0.0349	0.1427
	F_6_	0.76 (0.77)	0.23 (0.22)	0.01 (0.01)	0.88	0.12	1200	0.0748	<0.0001*
	F_7_	0.77 (0.77)	0.22 (0.22)	0.01 (0.01)	0.88	0.12	1440	0.1138	0.2772
	F_8_	0.79 (0.79)	0.20 (0.20)	0.01 (0.01)	0.89	0.11	900	0.5199	<0.0001*
	F_9_	0.83 (0.83)	0.17 (0.16)	0 (0.01)	0.92	0.08	560	0.0279	0.0252
	F_10_	0.78 (0.78)	0.21 (0.21)	0.01 (0.01)	0.89	0.11	480	0.4884	<0.0001*
	F_11_	0.36 (0.36)	0.49 (0.48)	0.15 (0.16)	0.61	0.39	520	0.5651	<0.0001*
	F_12_	0.38 (0.38)	0.48 (0.48)	0.14 (0.14)	0.62	0.38	240	0.7719	0.3329
									
B #1	F_2_	0.25	0.50	0.25	0.50	0.50			
	F_4_	0.35 (0.44)	0.62 (0.45)	0.03 (0.11)	0.66	0.34	160	<0.0001*	<0.0001*
	F_5_#	0.25 (0.36)	0.70 (0.48)	0.05 (0.16)	0.60	0.40	320	<0.0001*	0.0144
	F_6_#	0.13 (0.19)	0.61 (0.49)	0.26 (0.32)	0.44	0.56	760	<0.0001*	1
	F_7_	0.19 (0.29)	0.69 (0.50)	0.12 (0.21)	0.54	0.46	1440	<0.0001*	<0.0001*
	F_8_#	0.22 (0.29)	0.64 (0.50)	0.14 (0.21)	0.54	0.46	800	<0.0001*	0.4023
	F_9_#	0.13 (0.27)	0.77 (0.50)	0.10 (0.23)	0.52	0.48	1640	<0.0001*	0.0209
	F_10_#	0.24 (0.35)	0.71 (0.48)	0.05 (0.16)	0.60	0.40	680	<0.0001*	0.1192
	F_11_	0.73 (0.75)	0.27 (0.23)	0 (0.02)	0.87	0.13	680	<0.0001*	<0.0001*
	F_12_#	0.64 (0.66)	0.35 (0.30)	0.01 (0.04)	0.82	0.18	880	<0.0001*	0.9999
									
B #2	F_2_	0.25	0.50	0.25	0.50	0.50			
	F_4_	0.36 (0.46)	0.63 (0.44)	0.01 (0.10)	0.68	0.32	300	<0.0001*	<0.0001*
	F_5_	0.15 (0.27)	0.74 (0.50)	0.11 (0.23)	0.52	0.48	880	<0.0001*	<0.0001*
	F_6_#	0.30 (0.37)	0.62 (0.48)	0.08 (0.15)	0.61	0.39	1440	<0.0001*	1
	F_7_	0.18 (0.30)	0.74 (0.50)	0.08 (0.20)	0.55	0.45	1440	<0.0001*	<0.0001*
	F_8_	0.47 (0.50)	0.48 (0.42)	0.05 (0.08)	0.71	0.29	520	0.0002*	<0.0001*
	F_9_	0.38 (0.40)	0.50 (0.47)	0.12 (0.13)	0.63	0.37	920	0.0279	<0.0001*
	F_10_#	0.50 (0.53)	0.45 (0.40)	0.05 (0.07)	0.73	0.27	1800	<0.0001*	1
	F_11_	0.67 (0.70)	0.33 (0.28)	0 (0.02)	0.84	0.16	1000	<0.0001*	<0.0001*
	F_12_	0.50 (0.56)	0.50 (0.38)	0 (0.06)	0.75	0.25	1700	<0.0001*	<0.0001*

^*a *^total number of 4^th ^instar larvae used in genotype monitoring assay

^*b *^Nominal p-values are reported for the probability that the data fit the null hypothesis of Hardy-Weinberg equilibrium (or genetic drift)

* indicates tests that remain statistically significant after Bonferonni correction over the entire experiment

# indicates generations that are out of Hardy-Weinberg equilibrium likely due to genetic drift

Across all replicates, there are multiple generations where the genotype frequencies are not in Hardy-Weinberg equilibrium indicating that selection or genetic drift are acting on these generations (Table 1). This is true for F_4 _through F_11 _in cross A#1; F_4 _through F_12 _in cross B#1; and F_4 _through F_12_, excluding F_9_, in cross B#2. Using the allele frequency data, the null hypothesis of frequency changes being due to genetic drift can not be rejected for F_6_, F_7_, F_9 _and F_10 _of cross A#1; F_4_, F_5_, F_7_, F_9 _and F_12 _of cross A#2; F_5_, F_6_, F_8_, F_9_, F_10 _and F_12 _of cross B#1; and F_6 _and F_10 _in cross B#2. However, many of the same generations that reject genetic drift are also out of Hardy-Weinberg equilibrium (Table 1). This means that despite being in a laboratory setting where many conditions are optimized (food, space, etc.), these populations were probably undergoing selection and not genetic drift (Fisher's combined probability, χ^2^_72 _= 417, *p *< 0.0001) since the generations are out of Hardy-Weinberg equilibrium (Fisher's combined probability, χ^2^_72 _= 492, *p *< 0.0001) and there was no assortative mating.

### Temephos exposure

**Figure 3:**
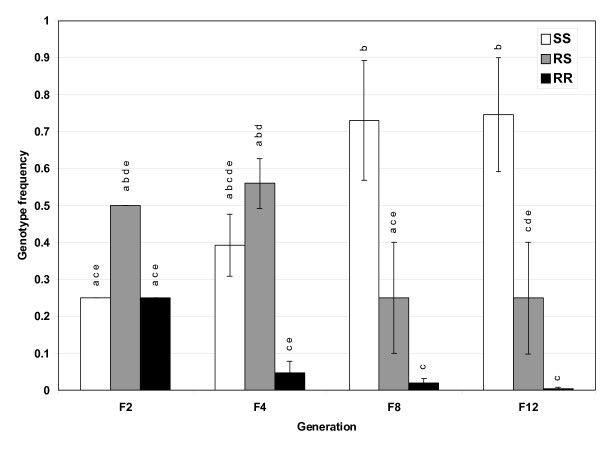
**P450 genotype frequencies in temephos exposed environment**. Frequencies of the P450-mediated detoxification genotypes monitored every 3 generations for a total of 12 generations in the temephos exposed environmental condition. Results are the average of four replicates. Bars are the S.E.M. Different letters indicate statistical differences (*p *< 0.05) between means.

The temephos exposed environment proved to be extremely detrimental to the maintenance of the R allele and RR genotype and more costly than the standard laboratory condition. The P450 detoxification R allele dramatically decreases from the F_2 _to the F_8 _generation where the frequency drops significantly to 0.13 from 0.5 (Figure 1). Between generations 8 and 12 the frequency plateaus, but given enough time, the R allele should be eliminated since at these two time points the R alleles are primarily in the heterozygotes (Table 2). The genotype pattern follows that expected from our original hypothesis. In the temephos exposed environment the RR genotype almost completely disappears, the RS genotype decreases from 0.5 (at F_2_) to 0.25 (at F_8 _and F_12_), while the SS genotype undergoes an increase in frequency (Figure 3) where two of the four cages become fixed for the S allele. In cage A#1, the RR and RS genotype are completely eliminated by generation F_4 _and F_12_, respectively and in cage A#2 by F_8 _and F_12_, respectively. The elimination of the R allele is more rapid in the A cross versus the B cross, though the B cross has a matching pattern where the R allele decreases through time and the RR genotype is eliminated from the population (Table 2). As expected, the temephos treated replicates are undergoing selection since they are out of Hardy-Weinberg equilibrium (Fisher's combined probability, χ^2^_24 _= 115, *p *< 0.0001) and this is not due to drift (Fisher's combined probability, χ^2^_24 _= 137, *p *< 0.0001). A previous study found the P450 present in ISOP450 bioactivates temephos, and that this P450 is tightly linked to (or identical to) the permethrin resistance allele [40]. This is likely one reason why the R allele in this environment is even more costly to the animal and is eliminated at a faster rate than the R allele in the standard laboratory environment.

**Table 2:** P450 genotype and allele frequencies in a temephos treated environment

		**Genotype frequency** **Observed (Expected)**			**Allele frequency**				
						
**Cross**	**Gen**.	**SS**	**RS**	**RR**	**S**	**R**	**n**^***a***^	**HWE**^***b***^	**Drift**^***b***^
									
A #1	F_2_	0.25	0.50	0.25	0.50	0.50			
	F_4_	0.44 (0.52)	0.56 (0.40)	0 (0.08)	0.72	0.28	180	<0.0001*	<0.0001*
	F_8_	0.98 (0.98)	0.02 (0.02)	0 (0)	0.99	0.01	560	0.8113	<0.0001*
	F_12_	1 (1)	0 (0)	0 (0)	1	0	220	1	0.2175
									
A #2	F_2_	0.25	0.50	0.25	0.50	0.50			
	F_4_	0.35 (0.45)	0.64 (0.44)	0.01 (0.11)	0.67	0.33	280	<0.0001*	<0.0001*
	F_8_	0.98 (0.98)	0.02 (0.02)	0 (0)	0.99	0.01	1640	0.6828	<0.0001*
	F_12_	1 (1)	0 (0)	0 (0)	1	0	440	1	0.2207
									
B #1	F_2_	0.25	0.50	0.25	0.50	0.50			
	F_4_	0.19 (0.28)	0.67 (0.50)	0.14 (0.23)	0.53	0.47	200	<0.0001*	0.2486
	F_8_	0.66 (0.66)	0.31 (0.30)	0.03 (0.03)	0.82	0.18	1060	0.3617	<0.0001*
	F_12_	0.60 (0.63)	0.39 (0.33)	0.01 (0.04)	0.80	0.20	1040	<0.0001*	0.3089
									
B #2	F_2_	0.25	0.50	0.25	0.50	0.50			
	F_4_	0.59 (0.60)	0.37 (0.35)	0.04 (0.05)	0.78	0.22	280	0.3078	<0.0001*
	F_8_	0.30 (0.39)	0.65 (0.47)	0.05 (0.14)	0.63	0.37	1640	<0.0001*	0.0052
	F_12_	0.38 (0.47)	0.61 (0.43)	0.01 (0.10)	0.69	0.31	80	0.0002*	0.1150

^*a *^total number of 4^th ^instar larvae used in genotype monitoring assay

^*b *^Nominal p-values are reported for the probability that the data fit the null hypothesis of Hardy-Weinberg equilibrium (or genetic drift)

* indicates tests that remain statistically significant after Bonferonni correction over the entire experiment

### Cold temperature environment

**Figure 4:**
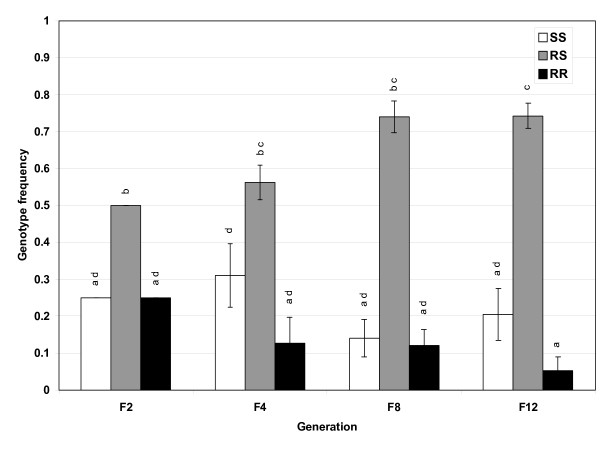
**P450 genotype frequencies in cold temperature environment**. Frequencies of the P450-mediated detoxification genotypes monitored every 3 generations for a total of 12 generations in the cold temperature environmental condition. Results are the average of four replicates. Bars are the S.E.M. Different letters indicate statistical differences (*p *< 0.05) between means.

In the cold temperature environment, no fitness cost of the P450 detoxification R allele frequency was observed. Despite frequency shifts between generations, the R allele stays in equilibrium around the 0.45 frequency level (Figure 1). While this equilibrium is slightly lower than the initial 0.5, the frequency at F_8 _is not statistically different from 0.5 (χ^2^_7 _= 0.816, *p *= 0.997). In three of the four cages (A#1, B#1 and B#2) the RR genotype decreases to frequencies of 0, 0.02 and 0.03, respectively. Surprisingly, there was a clear heterozygote advantage which allowed the R allele to persist in the cold temperature treated population (Figure 4). The SS genotype remains at frequencies close to the F_2 _starting frequency, ending in the F_12 _generation at 0.32, 0.27 and 0.23 for cages A#1, B#1 and B#2, respectively. Despite the A#2 cage RR frequency in generation 12 being 0.16 and the SS genotype frequency being 0, there was still a heterozygote advantage evident in this cage (Table 3). Almost all (11 of 12) of the monitored generations are out of Hardy-Weinberg equilibrium (Fisher's combined probability, χ^2^_24 _= 203, *p *≤ 0.0001) and the null hypothesis that drift is acting on the generations was rejected (Fisher's combined probability, χ^2 ^_24 _= 99, *p *< 0.0001) (Table 3), indicating that cold selection is driving the changes in genotype and allele frequencies.

**Table 3:** P450 genotype and allele frequencies in a cold selection environment

		**Genotype frequency** **Observed (Expected)**			**Allele frequency**				
						
**Cross**	**Gen**.	**SS**	**RS**	**RR**	**S**	**R**	**n**^***a***^	**HWE**^***b***^	**Drift**^***b***^
									
A #1	F_2_	0.25	0.50	0.25	0.50	0.50			
	F_4_	0.46 (0.53)	0.54 (0.39)	0 (0.08)	0.73	0.27	280	<0.0001*	<0.0001*
	F_8_	0.26 (0.38)	0.71 (0.47)	0.03 (0.15)	0.62	0.38	920	<0.0001*	0.0066
	F_12_	0.32 (0.44)	0.68 (0.45)	0 (0.11)	0.66	0.34	160	<0.0001*	0.1841
									
A #2	F_2_	0.25	0.50	0.25	0.50	0.50			
	F_4_	0.21 (0.21)	0.49 (0.49)	0.30 (0.30)	0.46	0.54	310	0.8320	0.1068
	F_8_	0.04 (0.17)	0.73 (0.48)	0.23 (0.35)	0.41	0.59	1280	<0.0001*	0.1716
	F_12_	0 (0.17)	0.84 (0.49)	0.16 (0.34)	0.42	0.58	1040	<0.0001*	0.3875
									
B #1	F_2_	0.25	0.50	0.25	0.50	0.50			
	F_4_	0.12 (0.22)	0.70 (0.50)	0.18 (0.28)	0.47	0.53	140	<0.0001*	0.2041
	F_8_	0.07 (0.28)	0.86 (0.50)	0.04 (0.22)	0.53	0.47	1160	<0.0001*	0.1204
	F_12_	0.27 (0.39)	0.71 (0.47)	0.02 (0.14)	0.63	0.37	560	<0.0001*	0.0284
									
B #2	F_2_	0.50	0.25	0.25	0.50	0.50			
	F_4_	0.45 (0.50)	0.52 (0.41)	0.03 (0.09)	0.71	0.29	270	<0.0001*	<0.0001*
	F_8_	0.19 (0.27)	0.66 (0.50)	0.15 (0.23)	0.52	0.48	840	<0.0001*	<0.0001*
	F_12_	0.23 (0.36)	0.74 (0.48)	0.03 (0.16)	0.60	0.40	800	<0.0001*	0.0529

^*a *^total number of 4^th ^instar larvae used in genotype monitoring assay

^*b *^Nominal p-values are reported for the probability that the data fit the null hypothesis of Hardy-Weinberg equilibrium (or genetic drift)

* indicates tests that remain statistically significant after Bonferonni correction over the entire experiment

## Discussion

Our results show that the fitness associated with cytochrome P450 monooxygenase-mediated permethrin resistance in *Cx. p. quinquefasciatus *varies according to the environment. We demonstrate a cost associated with this P450 detoxification mechanism when in competition with a susceptible allele under standard rearing conditions, and a greater cost when exposed to temephos. We also reveal a heterozygote advantage when in the cold temperature environment.

While a fitness cost was observed in the standard and temephos condition, no overall cost (decrease in frequency) of the R allele for this P450 locus was observed in the cold temperature environment. Intriguingly, all four replicates in the cold environment exhibited an excess of the heterozygote genotype. The RR genotype in all replicates did poorly, indicating that having two R alleles is costly. A similar pattern was shown in *Tribolium castaneum *where the susceptible allele was placed in direct competition with an allele that conferred malathion resistance (R^*mal*^). Over the 10 generations of the experiment, malathion resistant strains had a heterozygote advantage and the best fitness [41]. Observations of heterozygote advantage are rare and this phenomenon may contribute to the maintenance of genetic variation in populations [42].

The P450 detoxification mechanism under temephos exposure was shown to be highly costly. In the temephos exposed environment, the RR genotype frequency was entirely eliminated in two replicates and decreased to an average frequency of 0.0475 by the F_4 _generation. Hardstone et al. (2007) demonstrated low (0.73-fold), but significant, levels of negative cross-resistance to temephos in ISOP450 [40], suggesting enhanced bioactivation of temephos to temephos-oxon in the resistant strain. This is likely one of the reasons underlying the fitness cost of the R allele in the temephos treated environment. This suggests that vector control efforts could use temephos in populations with this P450 mechanism because it may serve as a method to slow the evolution of permethrin resistance. In a temephos treated environment, a population with this P450 will not be favored and a shift towards susceptibility at this locus will occur.

Many fitness studies on insecticide resistant insects have shown that resistance mechanisms (metabolic and target site) are costly [9,13,16,43,44]. However, no studies have looked at the fitness associated with P450-mediated resistance in mosquitoes. In this experiment we examined the fitness of the P450 allele under multiple environmental conditions, whereas several previous population cage studies limited the environmental condition to a relaxation of the insecticide selection pressure [45-47]. For example, the R allele in a strain of *Cx. p. quinquefasciatus *which was resistant to organophosphates due to elevated levels of Esterase-2 declined steadily when left unselected for 3 generations [45].

Population cage studies do not always show that a fitness cost is associated with the insecticide resistance allele. Examination of malathion resistance alleles in *Tribolium castaneum *under no known selection (including insecticide) pressure in a laboratory population cage experiment revealed the R allele is not costly and the frequency of this allele remained steady through time, independent of the starting R allele frequency [20]. Fluctuations in the R allele frequency from generation to generation were concluded to be the result of random genetic drift [20].

Very few studies have examined what happens to the R allele in a population cage study under sub-optimal environmental conditions. Raymond et al. (2005) placed two strains of diamondback moth (*Plutella xylostella*) with the same resistance mechanism conferring resistance to *Bacillus thuringiensis *in two different stressful environments in the lab. The two strains exhibited different fitness costs relative to each other under the same pressure and the fitness cost varied in each environment for an individual strain [21]. In contrast, no fitness costs were seen for diazinon resistant Australian sheep blowfly (*Lucilia cuprina*) when reared under three different larval densities or in a population cage competition experiment [48].

Resistance mechanisms due to metabolic processes are generally regarded as costly [49,50]. Some studies have proposed that the extent to which a metabolic resistance mechanism is costly depends on the relative expression of the protein in the resistant strain versus the susceptible strain [14]. Insecticide resistance due to gene amplification or gene duplication of general esterases is highly costly due to the resulting amount of enzyme that is produced. For example, in highly resistant green peach aphids, esterase amounts to approximately 3% of the total body protein [51]. Contrastingly, costs associated with increased monooxygenase activity have been found to be modest or absent. This phenomena has been shown using biotic potential measures in the house fly [52], laboratory, glasshouse and field studies measuring life-table parameters, predator-prey interactions, and mating competitiveness of the predatory mite, *Metaseiulus occidentalis *[53,54], in *Heliothis zea *after feeding on a diet with a P450 inducer and measuring food utilization parameters [55], and with P450-mediated resistance to DDT in *Drosophila melanogaster *[56]. Understanding the fitness costs associated with P450-mediated resistance is further complicated because even when the same insecticide is used, there can be plasticity in which P450(s) are selected for in different populations [57]. This could lead to variation in fitness costs between populations, due to the different P450s conferring the resistance.

## Conclusion

Determining the cost of resistance is imperative for implementing successful vector control strategies. As evident from the literature and our results, resistance alleles as well as genotypes are able to perform differentially (manifest varied levels of cost) in different environments. Additionally, in accordance with previous studies on insecticide resistance mechanisms, a fitness cost is associated with this P450 locus in an insecticide-free environment. It is also possible that differences present in the genetic backgrounds among the crosses could affect the fitness of the resistance allele. Understanding how resistant pest populations evolve under a spectrum of environmental conditions is essential if resistance management tactics are to be successful. Results from this study have implications for mosquito control strategies. For example, rotating permethrin and temephos use should slow the evolution of P450-mediated permethrin resistance. Additionally, the permethrin R allele can be maintained within the cold upper boundary of the *Cx. p. quinquefasicatus *habitat, where a hybrid region with *Cx. p. pipiens *is present. Therefore, the *Cx. p. quinquefasicatus *P450 R allele could be introduced into *Cx. p. pipiens *populations and have a negative impact on the control of this species.

## Methods

### Mosquito strains and insecticides

Three strains of *Culex pipiens quinquefasciatus *Say were used. SLAB is a standard susceptible strain [58]. JPAL is a strain highly resistant to permethrin due to *kdr *and cytochrome P450 monooxygenase-mediated (P450) detoxification [59]. ISOP450 was isolated by backcrossing JPAL into SLAB for 14 generations and selecting backcross progeny with permethrin. ISOP450 is a permethrin resistant strain (1,300-fold) containing only the cytochrome P450 monooxygenase mechanism of JPAL. P450-mediated resistance in ISOP450 is monofactorially inherited, incompletely dominant, autosomally linked, and larval specific [40].

All colonies were reared by standard methods. Larvae were reared with ample development space in plastic trays containing 2 L of distilled water for every 400 larvae. Larvae were provided abundant food consisting of a mixture of ground TetraFin^® ^goldfish flakes, rabbit pellets and liver powder (1:2:1) in distilled water. Adults were provided a 20% sugar solution *ad libitum*, and provided a chicken for 30 minutes two times per week (Cornell University Animal Use Protocol #01-56). All life stages were maintained at 27 ± 1°C, 80% relative humidity, and photoperiod of 14 hr:10 hr (L:D) including 2 hr of simulated dawn and 2 hr simulated dusk. Permethrin (98%) and temephos (98%) were obtained from Chem Service (Westchester, PA).

### Selection line cages for fitness analysis of P450-mediated resistance in mosquitoes

Selection line cages were initiated by randomly collecting at minimum 35 egg rafts from the main colony cages and rearing larvae under standard conditions. Pupae from each strain were individually isolated into plastic tubes. Upon eclosion, the respective sex and strain were released into a cage simultaneously to create the desired crosses. The adults were released into a cage *en masse *with an approximately 3:1 (female:male) ratio, with at least 200 females used. The following reciprocal crosses were created: SLAB females × ISOP450 males (referred to as cross A), and ISOP450 females × SLAB males (referred to as cross B). Crosses were reared under standard conditions. The F_2 _generation of both reciprocal crosses was then split into two cage replicates (referred to as #1 and #2) for each of the three environmental conditions (see below).

### Fitness of the P450-mediated permethrin resistance locus under standard laboratory conditions with no exposure to insecticides or other known selective pressures

In the standard laboratory environment, mosquitoes were reared under standard procedures with no exposure to insecticides or other known selective pressures which resulted in less than 1% mortality. At every generation, pupae were individually isolated so that virgin adults would be released into a new cage (minimum of 200 females and ratio of 2:1 females to males) within the same week and genotype frequencies were measured (see below). This cycle continued for a total of 12 generations.

### Fitness of the P450-mediated permethrin resistance locus under an environmental condition of temephos exposure

A temephos treatment generation occurred simultaneously for each replicate cage and consisted of a minimum of 300 larvae and an average of 1,600 larvae undergoing temephos selection. To apply the temephos stress, batches of 20 fourth instar larvae were placed in 4 oz. wax coated paper cups (Sweetheart Cup Co., Owings Mills, MD) with 99 ml of distilled water and 1 ml of temephos solution. The temephos concentration used throughout this experiment (0.0003 mg/ml) resulted in 60% mortality of the F_2 _generation. Control cups with 1 ml of acetone in 99 ml of distilled water were treated at the same time as treatment cups to insure that death in the treatment cups was due to temephos exposure. All cups were held at 25°C and larval mortality was assessed after 24 hours. Surviving treated larvae were then washed twice in clean distilled water and transferred to clean cups with 98 ml of distilled water and 2 ml of larval food. Surviving treated pupae were washed twice in clean distilled water and directly placed into individual tubes. Adults that emerged were released into a new cage that was placed in the standard rearing chamber. The succeeding generation to a treatment generation was started with a minimum of 100 surviving adult females and ratio of 2:1 females to males. Temephos treatment was performed for three consecutive generations. Genotype frequencies were measured (see below) every fourth generation and this generation would not undergo a temephos treatment. This cycle continued for a total of 12 generations.

### Fitness of the P450-mediated permethrin resistance locus under an environmental condition of cold temperature

A cold treatment generation occurred simultaneously for each replicate cage and consisted of a minimum of 600 larvae and an average of 1,300 larvae undergoing cold stress. To apply the cold temperature condition, batches of 20 fourth instar larvae were placed in 4 oz. wax coated paper cups with 98 ml of distilled water and 2 ml of larval food. The cups were then placed in a 16°C incubator for 4 consecutive days with a photoperiod of 14 hr:10 hr (L:D). These conditions resulted in an average of 60% mortality for each treatment generation. Simultaneously, cups serving as treatment controls were placed in a room temperature incubator (25°C) with a photoperiod of 14 hr:10 hr (L:D) to insure handling and food availability were not the cause of mortality in the treatment. On day 4, cold-treated pupae were place into individual tubes to complete development to adult at 25°C. Adult emergence rates were recorded in the treatment and control cups to determine mortality (*i.e*., selection pressure). The succeeding generation to a treatment generation was started with a minimum of 100 surviving adult females and ratio of 2:1 females to males. Cold treatment was performed for three consecutive generations. Genotype frequencies were measured (see below) every fourth generation and this generation would not undergo a cold treatment. This cycle continued for a total of 12 generations.

### Measuring genotype frequencies

**Figure 5:**
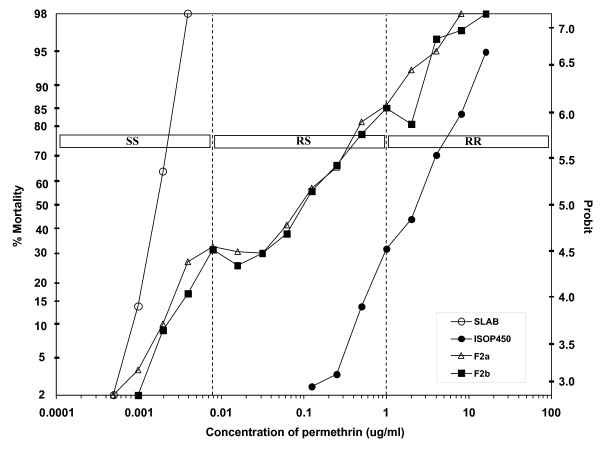
**Concentration-mortality lines of parental strains (SLAB and ISOP450) and the F_2 _generation of the reciprocal crosses of *Cx. p. quinquefasciatus***. Vertical dashed lines indicate the diagnostic concentrations used to distinguish the SS, RS and RR genotypes (shown in boxes).

Genotype frequencies were determined by performing bioassays on fourth instar larvae. The diagnostic concentrations to ascertain genotype frequencies were determined from the concentration-mortality curve of the F_2 _generation (Figure 5). The plateau at approximately 25% mortality (0.0078 ug/ml permethrin) clearly distinguished susceptible homozygotes (SS) while the plateau at approximately 75% mortality (1 ug/ml permethrin) separated SS and heterozygotes (RS) from the resistant homozygotes (RR).

For each larval bioassay, batches of 20 fourth instar larvae were placed in 4 oz. wax coated paper cups with 99 ml of distilled water and 1 ml of permethrin solution. Half of the available larvae were treated at each of the diagnostic permethrin concentrations such that mortality at 0.0078 ug/ml indicated the frequency of SS genotypes, survivorship at 1 ug/ml specified the frequency of RR genotypes and RS genotype frequencies were determined by taking 1 – SS genotype frequency – RR genotype frequency. The total number of larvae used ranged from 80–1,800 (Tables 1, 2 and 3) depending on the number of eggs laid in a cage. Test insecticide dilutions were dissolved in acetone and control cups were treated with 1 ml of acetone in 99 ml of distilled water. Mortality was assessed after 24 hours, and larvae were considered dead if they failed to move or resurface after being probed.

### Data analysis

Genotype frequencies were inferred from arc-sin transformed concentration dependent mortalities. Expected genotype frequencies (Table 1, 2 and 3) were calculated by using the observed allele frequencies from the previous generation. In all environments at all generations where genotype frequencies were measured, deviations from Hardy-Weinberg equilibrium were analyzed with a χ^2 ^test (*p *< 0.05). To account for multiple testing, we used the Bonferroni correction. Using Fisher's combined probabilities test we determined a global *p*-value for the set of Hardy-Weinberg equilibrium tests.

The probability of R allele frequency changes due to genetic drift acting alone between generations was calculated from the binomial distribution. For the temephos and cold environments, the probability of R allele frequency changes due to drift over the 2 and 4 generation intervals were simulated using R . The simulation assumed a panmictic population with fixed size of 200 diploid individuals with allele frequencies defined by the empirical observation at the start of the interval. For each generation, half of the alleles from the population were sampled at random and from this group the next generation allele frequencies were determined. This process was repeated for the number of generations within the interval of interest. *P*-values were obtained by dividing the number of simulations that resulted in an allele frequency change as great as or greater than the observed R allele frequency at the end of the interval of interest by the 10,000 total simluations run. The average of five *p*-values was reported and subjected to Bonferroni correction. Fisher's combined probabilities test was used to determine a global *p*-value for the set of genetic drift tests.

For the determination of R allele frequency changes, all crosses and replicates of an environmental condition were combined (n = 4). Statistical differences between means were determined using Tukey's test (*p *< 0.05).

## Authors' contributions

MCH and JGS conceived of and designed the study. MCH conducted the experiments and drafted the manuscript. BPL, MCH and JGS participated in statistical analysis, data interpretation and revisions of the manuscript. All authors read and approved the final manuscript.
